# Offline Parietal Intermittent Theta Burst Stimulation or Alpha Frequency Transcranial Alternating Current Stimulation Has No Effect on Visuospatial or Temporal Attention

**DOI:** 10.3389/fnins.2022.903977

**Published:** 2022-06-14

**Authors:** Jessica Moretti, Welber Marinovic, Alan R. Harvey, Jennifer Rodger, Troy A. W. Visser

**Affiliations:** ^1^School of Biological Sciences, The University of Western Australia, Perth, WA, Australia; ^2^Perron Institute for Neurological and Translational Science, Perth, WA, Australia; ^3^School of Population Health, Curtin University, Perth, WA, Australia; ^4^School of Human Sciences, The University of Western Australia, Perth, WA, Australia; ^5^Lions Eye Institute, Perth, WA, Australia; ^6^School of Psychological Science, The University of Western Australia, Perth, WA, Australia

**Keywords:** rTMS, iTBS, transcranial alternating current stimulation (tACS), attention, line bisection, attentional blink

## Abstract

Non-invasive brain stimulation is a growing field with potentially wide-ranging clinical and basic science applications due to its ability to transiently and safely change brain excitability. In this study we include two types of stimulation: repetitive transcranial magnetic stimulation (rTMS) and transcranial alternating current stimulation (tACS). Single session stimulations with either technique have previously been reported to induce changes in attention. To better understand and compare the effectiveness of each technique and the basis of their effects on cognition we assessed changes to both temporal and visuospatial attention using an attentional blink task and a line bisection task following offline stimulation with an intermittent theta burst (iTBS) rTMS protocol or 10 Hz tACS. Additionally, we included a novel rTMS stimulation technique, low-intensity (LI-)rTMS, also using an iTBS protocol, which uses stimulation intensities an order of magnitude below conventional rTMS. Animal models show that low-intensity rTMS modulates cortical excitability despite sub-action potential threshold stimulation. Stimulation was delivered in healthy participants over the right posterior parietal cortex (rPPC) using a within-subjects design (*n* = 24). Analyses showed no evidence for an effect of any stimulation technique on spatial biases in the line bisection task or on magnitude of the attentional blink. Our results suggests that rTMS and LI-rTMS using iTBS protocol and 10 Hz tACS over rPPC do not modulate performance in tasks assessing visuospatial or temporal attention.

## Introduction

Non-invasive brain stimulation is a growing field with wide clinical and basic science applications due to its ability to transiently and safely change brain excitability and oscillatory activity (Dayan et al., 2013; Lefaucheur et al., 2020). Research has shown that several brain stimulation techniques, including repetitive transcranial magnetic stimulation (rTMS) and transcranial electrical stimulation (tES), can modulate cognition in patients or healthy individuals by facilitating or disrupting mental processes. This has been suggested to arise *via* multiple potential mechanisms including induction of action potentials, changes to membrane potential and entrainment of endogenous brain oscillations (Miniussi et al., 2013). In particular, longer-term offline plastic changes are thought to be facilitated with rTMS through simultaneous depolarisation of pre- and post-synaptic neurons (Lenz et al., 2015), likely through rTMS induction of action potentials. However, brain stimulation in rodent models using a novel low-intensity (LI-) rTMS technique has shown that stimulation delivered at intensities below the action potential threshold (1–150 mT) can also induce behavioural and cellular changes, suggesting that direct induction of action potentials may not be necessary to induce such changes (Moretti and Rodger, 2022). Transcranial electrical stimulation also uses sub-action potential threshold stimulation and is able to induce various neuromodulatory effects on motor and cognitive function (Kuo and Nitsche, 2012; Flöel, 2014). Therefore LI-rTMS may be an intermediate approach combining the high focality of rTMS and the lower intensity stimulation of tES.

Low-intensity stimulation has several potential benefits including fewer side effects (e.g., headaches), reduced power requirements and the potential for more compact and portable design. LI-rTMS allows for these benefits while maintaining the focality of rTMS making it a desirable tool for translation. However, unlike conventional rTMS [which we will refer to as high-intensity (HI-) rTMS] and transcranial alternating current stimulation (tACS), LI-rTMS has not previously been used in humans, although there have been studies with sub-threshold pulsed magnetic fields, which is similar to LI-rTMS, that showed low intensity stimulation could modulate mood in humans (Rohan et al., 2004, 2014; Martiny et al., 2010). To explore LI-rTMS effects in humans for the first time, we included a LI-rTMS condition and assessed whether it could influence cognition.

We also aimed to compare LI-rTMS alongside HI-rTMS and tACS to explore the effects of different brain stimulation techniques in neuromodulation. There are not many studies in the literature which combine rTMS and tES in the same experiment to allow for direct comparisons between stimulation techniques. Single-session stimulation with HI-rTMS and tACS has previously been reported to induce cognitive change, including various aspects of attention (for reviews see Luber and Lisanby, 2014; Santarnecchi et al., 2015; Reteig et al., 2017). We test LI-rTMS in contrast with HI-rTMS, which has similar focality and includes a magnetic field, and tACS, which, like LI-rTMS, is a subthreshold stimulation, but uses widespread electrical, alternating current stimulation applied directly to the scalp. tACS was chosen as a comparative tES technique in order to match the alternating frequency and biphasic waveform of rTMS, as opposed to direct current stimulation. Therefore in this study, we assessed the impact of LI-rTMS, HI-rTMS, and tACS on human visuospatial attention in a within-subject design to compare relative efficacy.

Another aspect of cognitive modulation is the frequency protocol used to induce effects. Theta burst stimulation (TBS) is a complex patterned rTMS frequency often used in studies, with bursts of 3 pulses at 50 Hz applied at a frequency of 5 Hz. The bursts can be applied continuously for a set time [continuous (c)TBS], or intermittently in 2 s periods at a rate of 0.1 Hz [intermittent (i)TBS] to produce effects that are generally inhibitory or excitatory, respectively. Applying cTBS or iTBS to induce motor excitability changes is more efficient compared to simple patterned rTMS protocols (1 Hz, 10 Hz, etc.). The short application time of TBS protocols (3 min) also makes it an attractive stimulation technique. Despite the short stimulation time, cortical excitability changes induced by iTBS and cTBS have been observed for up to 60 and 50 min, respectively, after stimulation ends (Wischnewski and Schutter, 2015). Several studies have explored the use of iTBS and cTBS in cognitive domains to determine whether it is similarly effective for neuromodulation, with mixed results (e.g., Esterman et al., 2017; Gan et al., 2019; Mariner et al., 2021; Schintu et al., 2021; Whybird et al., 2021). We explore whether HI- and LI-rTMS applied using iTBS protocol are effective in enhancing visuospatial attention. We chose to assess attention as it is a higher-order cognitive process (Posner and Petersen, 1990) with several levels of processing susceptible to modulation by brain stimulation. Attention collectively refers to processes involved in the selection of environmental information to support behaviour. Here we focus on two of these processes–spatial and temporal attention–which are used to direct cognitive resources to specific locations in space or specific periods of time. We assessed participants’ spatial attention using the line bisection (Landmark) task and temporal attention with an attentional blink (AB) task across three sessions with different stimulation types.

The stimulation site, over the rPPC, was kept consistent between groups with the Cz as the reference electrode with tACS. We hypothesised that excitatory offline HI- and LI- rTMS over the right posterior parietal cortex would induce a leftward shift in spatial bias in the line bisection task and reduce the attentional blink in the AB task in line with previous studies (e.g., line bisection: Fierro et al., 2000; Hilgetag et al., 2001; Kim et al., 2005; Thut et al., 2005; Nyffeler et al., 2008; attentional blink: Cooper et al., 2004). In contrast, alpha frequency (10 Hz) is associated with inhibition of visual perception and attention, therefore alpha frequency tACS is thought to inhibit visual attention (Jensen and Mazaheri, 2010; Foxe and Snyder, 2011; Clayton et al., 2015; c.f. Clayton et al., 2019). Therefore, we hypothesised that rPPC tACS would induce a rightward shift in spatial bias and inhibit temporal attention, possibly increasing the attentional blink.

## Materials and Methods

This study was approved by the University of Western Australia Human Research Ethics Committee (RA/4/20/6005) and all participants gave informed consent. Twenty-four participants (15 female, 9 male, all self-reported as right-handed, mean age = 19.5 years, *SD* = 2.7) with normal or corrected-to-normal vision participated in the study. The exclusion criteria used for selection conformed to the guidelines for rTMS (Rossi et al., 2009) and tES research (Antal et al., 2017). Participants were undergraduate university students and received partial course credit in exchange for their participation. Four participants withdrew from the experiment: one without an explanation, one due to an injury between sessions affecting their vision and two due to adverse side effects following HI-rTMS session. Adverse side effects included a headache for one participant and “tightness” in the jaw for the other, possibly in reaction to the repeated tapping sensations. Available data from these participants from previous sessions with no adverse effects were still included.

**FIGURE 1:**
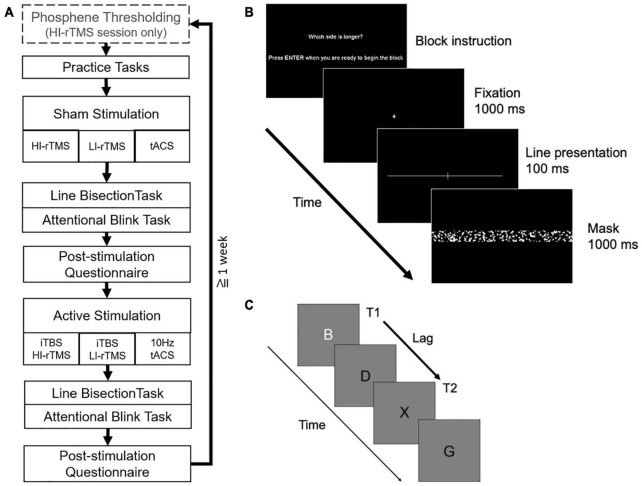
**(A)** Overall order and structure of each experimental session. **(B)** Example of a trial in the line bisection task. **(C)** Example letter sequence in the attentional blink task (T2 shown at lag 2 position).

Participants received three types of stimulation (HI-rTMS, LI-rTMS, or tACS) in separate sessions (counterbalanced) separated by at least a week to prevent carry-over effects using a cross-over, within-subject design. In order to minimise the number of repeat visits required and increase retention, participants received both sham and active stimulation in each session. Sham was delivered first to avoid carry-over effects of stimulation. Participants were informed that they would receive both sham and active stimulation each session but were blinded to the order. Each session followed the same sequence (Figure 1A). For the HI-rTMS session, there was an additional thresholding step at the beginning of the session to determine the participant’s phosphene threshold.

A post-stimulation questionnaire was administered to assess for possible side-effects and whether participants thought they received a sham or active stimulation. All experiments were run on a Windows computer using specialised software programmed in PsychoPy (Peirce et al., 2019). Stimuli were presented on a 24-inch monitor running at a refresh rate of 60 Hz with a viewing distance of 55 cm.

### Line Bisection Task

The line bisection task was similar to Kim et al. (2005; see Figure 1B). Stimuli were white, horizontal lines transected or bisected by a white 2.2 degree vertical line on a black background. All lines were 0.1 degree thick. The horizontal line was one of 5 lengths (36–40 degrees) with each length presented equally often. When the horizontal line was transected the elongated side was longer by 1 degree, and the vertical transecting line remained in the centre of the screen.

A single trial consisted of a fixation cross which appeared for 1000 ms followed by a line stimulus presented for 100 ms. The line stimulus was then masked for 1000 ms by a noise mask (50.6 degrees × 20.92 degrees) consisting of randomly generated white or grey solid circles of various sizes. Before each block, participants were instructed to report either which side of the line was longer or which side was shorter. The question alternated each block, and each task alternated which question began the first block. Participants were instructed to respond quickly without sacrificing accuracy by pressing the left and right arrow keys with their right index and middle finger, respectively. If participants did not respond within 1000 ms of mask onset, the trial was considered an error and the next trial was initiated.

Prior to each session, two blocks of 30 practice trials were completed, each consisting of 15 left-elongated, and 15 right-elongated lines presented for 200 ms to allow participants to familiarise themselves with the task. This was followed by the main task consisting of four blocks of 40 trials (10 lines transected with left-side elongation, 10 lines transected with right-side elongation, 20 evenly bisected lines presented in random order).

### Attentional Blink Task

The Attentional Blink task was similar to Cooper et al. (2004; see Figure 1C). Letter stimuli were presented in black, 48 pt Helvetica font on a grey background. A single trial consisted of a fixation cross presented for 1000 ms followed by a stream of 17 letters presented for 20 ms each with an 80 ms blank inter-letter interval. The first target (T1) was a white letter that could appear randomly in positions 4, 5, 6, 7, or 8 in the stream. The second target (T2) was a black letter X that could appear 1, 2, 3, 5, or 7 positions (lags) after the white letter. The white letter (T1) was chosen from a subset of letters: N, Z, B, E, L, T, W, and M. Non-target letters were chosen from the remaining letters of the alphabet (except X). After the stream was complete, participants were prompted to report the identity of the white letter by pressing the matching key with their left hand and to report whether there had been an X presented by pressing marked arrow keys with their right hand. Participants were instructed to emphasise accurate responding. Following participant responses, there was a 500 ms blank interval before a new trial began.

The experimental tasks consist of 2 blocks of 55 trials. Forty trials included T2, presented equally often at each lag. The order of the trials were randomised for each block. Before beginning the task, participants were told that at least 50% of the trials contained an X, in order to reduce a bias towards reporting the absence of T2. Prior to beginning the experimental task, participants completed two blocks of 125 trials as practice to thoroughly familiarise themselves with the task requirements.

### Stimulation

#### Determining Phosphene Threshold

For rTMS we used the MagPro R30 Stimulator (Magventure, Denmark) with a 75 mm Figure-of-8 coil (MC-B65-HO-2). At the beginning of the session single TMS pulses are delivered to the back of the head under dim lighting to determine the phosphene threshold of the participant based on the methods of Kammer et al. (2001). We conduct a searching procedure for a phosphene “hot spot” over the right hemisphere, beginning 3 cm dorsal and 5 cm lateral from the inion. We deliver single TMS pulses at a high intensity [up to 80% maximum stimulator output (MSO)] and systematically move the coil until the participant reliably reports seeing phosphenes following the TMS. Once the hot spot is located, we adjust the TMS intensity down in steps of 5%, and then 1% MSO, delivering 10 consecutive pulses at each intensity level. The lowest intensity at which 5 out of 10 pulses are reported to induce a phosphene in the participant’s vision is determined to be the phosphene threshold. If a participant failed to reliably see phosphenes, stimulation at 50% MSO for HI-rTMS was used, or the next highest intensity that was comfortable for the participant.

#### Repetitive Transcranial Magnetic Stimulation

For the rTMS stimulation we delivered 600 pulses (biphasic sine waves, 3 min) using the iTBS protocol at either 90% phosphene threshold [34–53% maximum stimulator output (MSO)] (HI-rTMS) or 7% MSO (LI-rTMS) over the right posterior parietal cortex (rPPC) (electrode site P4). Seven percent MSO was equivalent to approximately 50 mT at the estimated distance of the cortical surface (2.5 cm from the scalp), based on magnetic field measurements from the coil. This intensity was chosen to match LI-rTMS parameters that have previously been delivered in animal models (Heath et al., 2018).

In each session participants received a sham and active stimulation. For the sham stimulation, the coil was set to 0% MSO and held above electrode site P4 by the experimenter, with a speaker playing a recording of the appropriate rTMS protocol to mimic the auditory sensation.

#### Transcranial Alternating Current Stimulation

**FIGURE 2:**
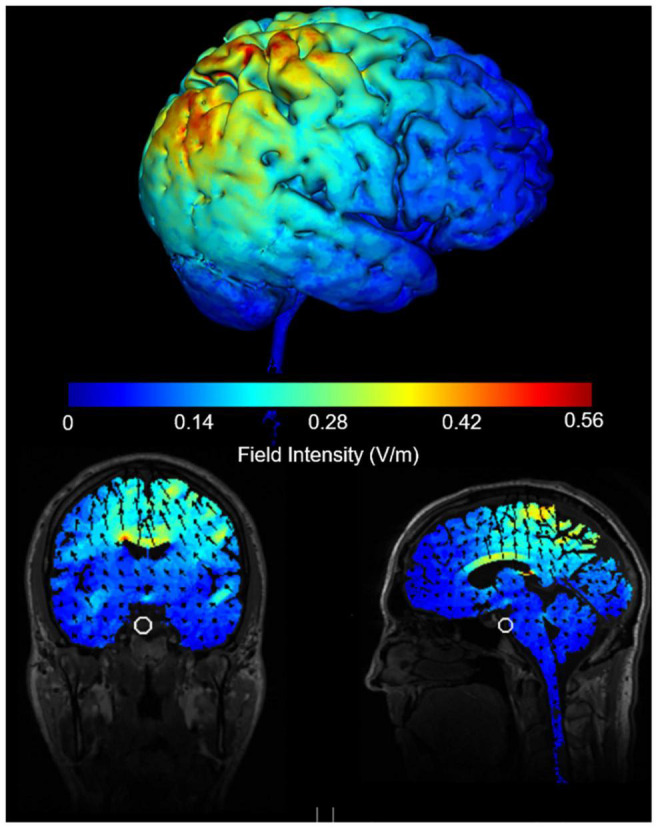
Induced e-field modelling of tACS parameters when electrodes are positioned at Cz and P4 and delivering 2 mA peak to peak intensity.

A multichannel neuromodulation system (Soterix Medical, United States, Model: MXN-5) was used to deliver 20 min (with 30 s ramp up/down) of 10 Hz tACS at 2 mA peak-to-peak amplitude (biphasic sine waves) to the rPPC. Two 5 × 7 cm rubber electrodes in saline-soaked sponges were placed above electrode sites P4 and Cz with electrode gel for added conduction and secured in place with bandages. There was no overlap between the two electrodes. The induced e-field produced with the electrode positioning was modelled using Soterix software (Figure 2). The Cz was chosen as the reference electrode based on previous tES studies that examined attention when stimulating rPPC (Sparing et al., 2009; Loftus and Nicholls, 2012; Filmer et al., 2015; Hopfinger et al., 2017). Participants received a sham and active stimulation. For sham stimulation, current was ramped up over 30 s and immediately ramped down over 30 s at both the beginning and end of stimulation.

## Results

### Data Analysis

For the AB task one participant was excluded due to self-reported inability to see T2 at any point during a session (*n* = 23).

Remaining data were analysed using generalised logistic mixed models at the trial level. For the line bisection task, bias scores were calculated by coding responses to bisected lines as “0” when the response indicated that the left side appeared longer, and “1” when the response indicated that the right side appeared longer.

### Line Bisection Task

#### Task Accuracy

Accuracy on the line bisection task for unevenly transected lines was analysed using generalised mixed model with fixed effects set as elongated side, Stimulation Type, and Active vs. Sham stimulation (see Table 1) and subject as a random effect. There was a significant main effect for Stimulation Type (χ^2^ = 9.54, *p* = 0.008), but no other significant effects or interactions (χ^2^ ≤ 5.44, *p* > 0.066). Follow up analyses indicated that accuracy during the HI-rTMS sessions was significantly lower compared to tACS sessions (*z* = −2.66, *p* = 0.008) and LI-rTMS sessions (*z* = −2.73, *p* = 0.006). However, accuracy during the tACS and LI-rTMS sessions did not differ (*z* = −0.06, *p* = 0.949).

**TABLE 1:** Mean accuracy (%) when responding to transected line stimuli in the line bisection task.

Stimulation type		Mean accuracy (SD) (%)	
		Left elongated	Right elongated
HI-rTMS	Sham	57.1 (5.0)	61.7 (4.9)
	Active	58.2 (4.9)	63.3 (4.8)
LI-rTMS	Sham	66.7 (4.7)	64.8 (4.8)
	Active	65.2 (4.9)	63.6 (4.8)
tACS	Sham	61.1 (4.9)	71.2 (4.5)
	Active	65.2 (4.8)	62.5 (4.9)

*Numbers in brackets represent standard deviation.*

#### Spatial Bias

##### Initial Bias

One-sample *t*-tests showed bias scores for evenly bisected lines during following sham stimulation were not significantly different from 0.5 for HI-rTMS and LI-rTMS sessions, but there was a slight but significant rightward bias for the tACS session [HI-rTMS: *M* = 0.4997, *t*(1484) = −0.026, *p* = 0.979; LI-rTMS: *M* = 0.4968, *t*(1716) = −0.265, *p* = 0.791; tACS: *M* = 0.5404, *t*(1657) = 3.30, *p* < 0.001]. This suggests none of the participant conditions showed the conventional leftward spatial bias (pseudoneglect) (Milner et al., 1992; Learmonth et al., 2015) prior to stimulation.

##### Effect of Stimulation

**FIGURE 3:**
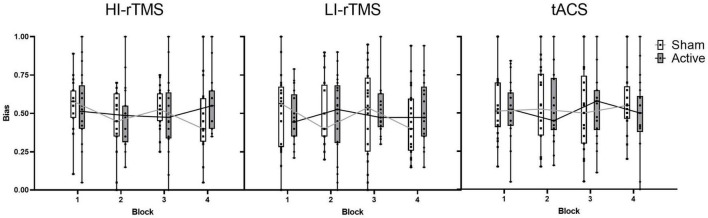
Spatial bias scores for sham and active stimulation for each stimulation type across blocks of the line bisection task. No significant effects or interactions present. Individual points represent mean spatial bias for individual participants. For the bias score: 0 = absolute leftward bias, 1 = absolute rightward bias.

Bias scores were analysed with a generalised linear mixed model with fixed factors of Stimulation Type, Active vs. Sham Stimulation and Block and subject included as a random effect (Figure 3). Block was included as a variable in order to assess for any delayed effects of stimulation (Gamboa et al., 2010; Gan et al., 2019). There was a significant main effect of Stimulation Type (χ^2^ = 14.9, *p* < 0.001), but no significant main effects of factors Active vs. Sham Stimulation (χ^2^ = 0.806, *p* < 0.369) or Block (χ^2^ = 4.89, *p* = 0.180). There was also a significant interaction for Stimulation Type*Block (χ^2^ = 12.8, *p* = 0.046) and Active vs. Sham Stimulation *Stimulation Type *Block interaction (χ^2^ = 15.9, *p* = 0.014). In order to understand the nature of the interaction, we followed up with simple effect comparisons, contrasting sham vs. active stimulation between the same block for each Stimulation Type (i.e., comparing Sham HI-rTMS Block 1 with Active HI-rTMS Block 1; see Table 2). There were significant effects for HI-rTMS block 3 and 4; LI-rTMS block 4 and tACS block 4, but none survived adjustment for multiple comparison using Holm–Sidak corrections. We also performed simple effect comparisons for the Stimulation Type*Block interaction, comparing stimulation types across each block. The only comparison that survived Holm–Sidak multiple comparison correction was a difference between HI-rTMS and tACS in block 1 (*z* = −2.99, *p* = 0.035). The simple effects also suggest that the main effect of Stimulation Type cannot be interpreted as there was no pairwise comparison between two stimulation types that was significantly different across all blocks. Thus, inter-session performance was relatively stable.

**TABLE 2:** Simple effect comparisons for the Active vs. Sham Stimulation *Stimulation type *Block interaction for spatial bias.

Block	Stimulation type	Contrast	*z*	*p*_unadjusted_	*p*_adjusted_
1	HI-rTMS	Active vs. Sham	−1.312	0.190	0.815
	LI-rTMS	Active vs. Sham	−0.793	0.428	0.955
	tACS	Active vs. Sham	−1.028	0.304	0.921
2	HI-rTMS	Active vs. Sham	0.147	0.883	0.986
	LI-rTMS	Active vs. Sham	−0.106	0.915	0.986
	tACS	Active vs. Sham	−0.602	0.547	0.958
3	HI-rTMS	Active vs. Sham	−2.028	0.043	0.356
	LI-rTMS	Active vs. Sham	−0.532	0.595	0.958
	tACS	Active vs. Sham	0.835	0.404	0.955
4	HI-rTMS	Active vs. Sham	2.433	0.015	0.166
	LI-rTMS	Active vs. Sham	1.970	0.049	0.364
	tACS	Active vs. Sham	−2.135	0.033	0.309

*Adjusted p-values use Holm–Sidak corrections for multiple comparison.*

### Attentional Blink

#### T1 Accuracy

T1 accuracy (Table 3) was analysed using generalised linear mixed model with Stimulation Type, Active vs. Sham stimulation and Lag included as fixed factors, and subject included as a random effect. As can be seen in the Table, overall accuracy was close to ceiling. Nevertheless, there was a significant main effect of Stimulation Type (χ^2^ = 9.87, *p* = 0.007) and a main effect of Active vs. Sham stimulation (χ^2^ = 3.86, *p* = 0.049), but no main effect of Lag (χ^2^ = 5.47, *p* = 0.361) and no significant interactions (χ^2^ ≤ 8.15, *p* > 0.258). Follow up comparisons indicated that accuracy during the HI-rTMS session (92.1% ± 1.47) was significantly higher compared to both LI-rTMS (90.4% ± 1.73; *z* = 2.97, *p* = 0.003) and tACS sessions (90.7 ± 1.69; *z* = 2.50, *p* = 0.0012). T1 accuracy during LI-rTMS and tACS sessions did not differ from each other (*z* = −0.479, *p* = 0.632). The difference between Sham and Active stimulation, although significant, was quite small, and not necessarily meaningful, with mean accuracy reduced by 1% following active stimulation (Sham T1 Accuracy: 91.6% ± 1.53; Active T1 Accuracy: 90.6% ± 1.68). The lack of an interaction effect with Stimulation Type also indicates that the stimulation effect was not specific to, or more pronounced for a particular stimulation technique.

**TABLE 3:** Mean accuracy (%) when responding to T1 in the attentional blink task.

Stimulation type		Mean accuracy (SD) (%)					
		T2 absent	Lag 1	Lag 2	Lag 3	Lag 5	Lag 7
HI-rTMS	Sham	89.5 (30.7)	90.8 (29.0)	91.1 (28.5)	88.5 (32.0)	88.8 (31.6)	91.1 (28.5)
	Active	89.4 (30.8)	91.7 (27.7)	86.5 (34.3)	90.6 (29.2)	87.2 (33.5)	88.9 (31.5)
LI-rTMS	Sham	88.5 (31.9)	89.4 (30.9)	89.1 (31.3)	89.1 (31.3)	87.5 (33.1)	87.2 (33.5)
	Active	87.1 (33.5)	88.4 (32.1)	86.0 (34.7)	84.2 (36.5)	87.5 (33.1)	84.8 (35.9)
tACS	Sham	86.5 (34.2)	87.8 (32.8)	89.3 (31)	90.2 (29.8)	86.3 (34.4)	86.3 (34.4)
	Active	90.2 (29.8)	86.3 (34.4)	87.8 (32.8)	87.8 (32.8)	87.2 (33.5)	84.2 (36.5)

*Numbers in brackets represent standard deviation. Accuracy for T1 was significantly lower with stimulation.*

#### T2|T1 Accuracy

**FIGURE 4:**
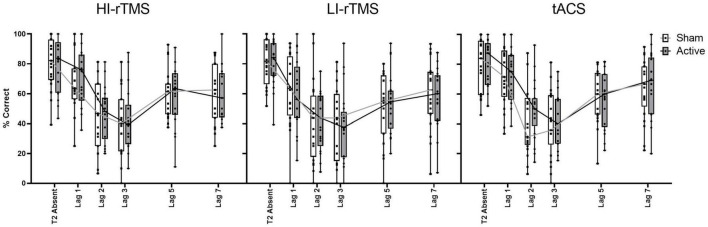
Percentage accuracy for reporting T2| T1 across lag positions for each stimulation type. The attentional blink occurred with Lag 1 sparing. Accuracy did not differ between stimulation parameters for T2| T1. Individual points represent mean accuracy for individual participants.

In order to assess the group effects of stimulation on temporal attention, T2 accuracy calculated only on trials when T1 is correct (T2|T1 Accuracy) was analysed using a generalised linear mixed model with fixed factors of Stimulation Type, Active vs. Sham Stimulation and Lag, with subject included as a random effect. There was a significant effect of Lag (χ^2^ = 986, *p* < 0.001) and a significant interaction with Stimulation Type * Lag (χ^2^ = 19.7, *p* < 0.032), indicating a robust attentional blink with Lag 1 sparing (Figure 4). There were no other significant main effects or interactions, χ^2^ ≤ 4.91, *p* > 0.092. The interaction between Stimulation Type * Lag suggest that there was some slight difference in attentional blink between sessions, but since there was no interaction with Active vs. Sham Stimulation, it is not connected with application of active stimulation.

### Sensation and Blinding During Stimulation

For HI-rTMS, 85% of participants correctly guessed when they received the sham stimulation, and 90% correctly guessed the active stimulation. For LI-rTMS, 57% correctly guessed the sham stimulation, but only 38% correctly guessed the active stimulation. For tACS, 41% correctly guessed the sham stimulation, while 36% correctly guessed the active stimulation. Tapping and tingling sensations were reported following HI-rTMS and tACS, respectively, in some participants. No physical sensation was reported following LI-rTMS. The HI-rTMS sham was not as effective as tACS or LI-rTMS, however, as there were no stimulation type effects in tasks it does not appear that there were disproportionate sham or expectancy effects.

## Discussion

In this study we assessed whether offline LI-rTMS or HI-rTMS delivering iTBS and 10 Hz tACS would induce shifts in visuospatial attention in a line bisection task and alter temporal attention in an AB task. Overall, offline brain stimulation did not change performance in either task, with the exception of a small reduction in T1 accuracy during the attentional blink task following active stimulation.

The lack of significant differences following stimulation in task performance related to attention was unexpected as several studies report changes to cognition following stimulation, particularly for the line bisection task (e.g., line bisection: Fierro et al., 2000; Hilgetag et al., 2001; Kim et al., 2005; Thut et al., 2005; Nyffeler et al., 2008; attentional blink: Cooper et al., 2004). On the face of it, one might speculate that the absence of stimulation effects may reflect the absence of pseudoneglect at a group level, potentially suggesting a lack of sensitivity to spatial bias. However, we think this explanation is unlikely for two reasons. First, our task was based on Kim et al.’s (2005) study, which showed a robust pre-stimulation leftward bias and thus should be sensitive to stimulation effects on spatial bias if present in our sample. Second, despite the lack of evidence for pseudoneglect at a group level, our statistical analyses accounted for individuals’ biases and their change over time to maximise statistical sensitivity to modulation. Notably, our other attentional task–the attentional blink–showed a robust group-level attention effect but also no changes in temporal attention following stimulation, making the suggestion that the absence of stimulation effects depends on the presence of group level attention affects prior to stimulation less plausible.

It is also possible that our study design, comparing task results following sham and active stimulation, may overlook effects elicited due to sham intervention. If such effects occurred it may have resulted in ceiling effects that active stimulation could not improve upon. For example, in a study of discrimination sensitivity, there was an instance of a sham intervention effect where active transcranial direct current stimulation (tDCS) stimulation compared against baseline resulted in a significant effect, but comparison against sham did not (Benwell et al., 2015). However, this pattern was not found for attentional bias which was examined in the same study. Moreover, other studies have included separate baseline vs. sham comparisons in similar cognitive tasks and not shown a significant sham intervention effect (Kim et al., 2005; Giglia et al., 2011; Learmonth et al., 2017). Therefore, we believe previous studies suggest that the contribution of a sham-elicited effect is unlikely in our design; however, this needs further investigation. Below, we discuss our results in relation to the current brain stimulation literature and consider possible contributing factors to our non-significant results in greater detail.

### Contributing Factors–Stimulation Protocols

#### Theta Burst Stimulation

We differed from several previous experimental designs in that we applied HI- and LI-rTMS delivering iTBS rather than a simple patterned rTMS protocol such as 10 or 1 Hz stimulation (e.g., Hilgetag et al., 2001; Kim et al., 2005). However, previous studies have shown iTBS and cTBS can induce cognitive effects (for review see Demeter, 2016). Specifically, after stimulation over the right parietal cortex, cTBS has been shown to induce spatial attention deficits in healthy participants (Nyffeler et al., 2008; Cazzoli et al., 2009; Rizk et al., 2013; Varnava et al., 2013; Chechlacz et al., 2015; Schintu et al., 2021) and alleviate deficits in neglect patients (Nyffeler et al., 2009; Cazzoli et al., 2015; Fu et al., 2015; Yang et al., 2015). cTBS stimulation was also more effective at alleviating deficits than simple patterned protocols for neglect patients (Fu et al., 2015). Cerebellar iTBS was also shown to improve performance in an AB task (Esterman et al., 2017), while cerebellar cTBS increased the AB (Arasanz et al., 2012). Unfortunately, iTBS over the PPC has not been extensively assessed in cognitive studies, although one recent study assessing TBS over the parietal cortex showed changes to inhibition, sequence learning and working memory, but not spatial attention in a simple cue task following both iTBS and cTBS (Whybird et al., 2021). Another recent study compared iTBS over the left PPC with sham, high definition-tDCS and a cTBS protocol in tasks assessing working memory, divided attention, and generalised attention (Stroop task) (Gan et al., 2019). All active stimulation conditions improved reaction times in the generalised attention task, but there was no significant effect on divided attention or working memory. iTBS also had the largest effect size, followed by tDCS and cTBS (Gan et al., 2019). Both Gan et al. (2019) and Whybird et al. (2021) show that iTBS can induce cognitive changes, however, it is not yet established which areas of cognition iTBS can reliably modulate. Whybird et al. (2021) demonstrated modulation of working memory, but Gan et al. (2019) showed no significant modulation of working memory. Despite the contrasting working memory results, both studies show reduced reaction time in inhibition related tasks following left PPC stimulation (emotional Stroop task: Gan et al., 2019; NoGo task: Whybird et al., 2021).

For this study we were interested in whether an iTBS protocol was able to be an effective cognitive enhancement tool. Although iTBS often has the opposing action to cTBS, it may be that iTBS over the rPPC does not induce the opposing behavioural effects evidenced by cTBS in previous spatial attention studies. Disruption of cognition also tends to be more easily induced than cognitive enhancement (Luber and Lisanby, 2014), which could explain the propensity for cTBS but not iTBS effects, especially if attention is already operating at high efficacy. Although when iTBS did appear to induce changes, it was more effective than cTBS (Gan et al., 2019). Compared to simple patterned protocols (i.e., 10 Hz, 1 Hz), iTBS can induce stronger and longer lasting effects compared to simple patterned protocols in measurements of synaptic plasticity (Huang et al., 2005) and has also been more effective than simple protocols in other cognition studies (Fu et al., 2015; Wu et al., 2021). However, this may not be the case for the tasks included in this study. The lack of significant changes to spatial attention in this study are in line with the lack of changes to attention cuing, a spatial attention task, seen in Whybird et al. (2021).

#### Transcranial Alternating Current Stimulation vs. Transcranial Direct Current Stimulation

We included offline tACS as a comparison with HI- and LI- rTMS in order to compare whether biphasic stimulation *via* application of alternating current directly onto the scalp would differ in strength of effect compared to magnetic stimulation. We theorised that differences could possibly provide information about differences in mechanisms between rTMS and tES, particularly between LI-rTMS and tES as both induce sub-action potential threshold levels of electrical stimulation. We therefore chose tACS for this study in order to have alternating current stimulation across all three stimulation types. One potential limitation of this choice, is that tDCS is more commonly used to induce cognitive effects. However, tACS has previously been shown to affect attention and various other forms of cognition (for review see Klink et al., 2020). For example, Yaple and Vakhrushev (2018) reported changes to temporal attention in an attentional blink task following 20 Hz tACS. Schuhmann et al. (2019) also reported a shift in spatial attention in cued attention and detection tasks with 10 Hz tACS and Otsuru et al. (2019) documented changes to spatial bias and temporal discrimination in a temporal order judgement task with 10 Hz tACS. Nonetheless, since there is less evidence for spatial and temporal attention modulation with tACS, it remains possible that tDCS could have been a more effective stimulation method.

##### Offline Stimulation

Another difference with many other tACS and tDCS studies is that we applied tACS offline, rather than online, in order to match the timing of rTMS stimulation and to facilitate a comparison between electrical vs. magnetic stimulation. However, a potential problem with this choice is that one of the main proposed mechanisms of tACS is its ability to entrain alpha wave brain oscillation during a cognitive task to induce cognitive effects (Dayan et al., 2013; Miniussi et al., 2013), and previous positive results used online stimulation (Yaple and Vakhrushev, 2018; Otsuru et al., 2019; Schuhmann et al., 2019). In addition, Veniero et al. (2017) initially showed that tACS during the line bisection task (online) but not preceding the task (offline) was able to shift spatial attention, although they could not replicate the result. That said, offline tACS can induce cognitive effects in other domains (e.g., memory and perception, see Klink et al., 2020 for review), and can enhance alpha oscillations, apparently *via* spike-timing dependent plasticity rather than direct entrainment (Vossen et al., 2015). Nonetheless, for future studies, if the aim is modulation of attention, it may be better to use online interventions.

#### Stimulation Methodology

An additional consideration is advancements in brain stimulation techniques which can refine stimulation protocols. For example, although the use of 10–20 EEG positions to target stimulation sites are quick and easy to administer, it has limited accuracy. A study comparing methods of determining stimulation sites found that the EEG coordinate approach using “P4” was associated with the lowest behavioural effect size in a number comparison task, while fMRI- and MRI-guided neuronavigation was most effective (Sack et al., 2009). Therefore using MRI-guided rTMS would allow for more precise and consistent stimulation site targeting which could lead to greater likelihood of significant stimulation findings. However, the need for MRI scanning and specialised equipment means this option is highly dependent on the resources available to the researcher. Furthermore, applying tACS using individual alpha frequency rather than fixed frequency may be a more successful way to induce tACS effects. Individual alpha frequency tACS has been associated with long-lasting after effects due to plastic changes (e.g., Vossen et al., 2015) and is increasingly the preferred method for applying tACS. However, comparisons between fixed and individualised alpha frequency tACS are still needed to compare the efficacy of the two techniques.

#### Stimulation Intensity

Another factor of stimulation is the intensity chosen for each stimulation. This is a source of variation across all brain stimulation studies, with no uniform approach, particularly since various brain regions may respond differently to differing intensities and “the more, the better” is often not the case. Without a specific dose-response curve, it is difficult to conclude whether the intensity dosage was optimal for each stimulation, however, selection of intensities reflected previous studies.

For HI-rTMS, iTBS is usually applied between 70 and 90% of an individual’s active or resting motor threshold (Turi et al., 2021). A limitation with regards to intensity comparisons is that we are in the minority as studies who use phosphene thresholds as a way to individualise stimulation intensities (Turi et al., 2021). There is some criticism for whether motor thresholds or phosphene thresholds are appropriate for guiding amplitude selection in non-motor or non-visual areas (Stewart et al., 2001; Boroojerdi et al., 2002; Beynel et al., 2019). Furthermore, a meta-analysis of online rTMS studies’ effects on cognition found that use of fixed versus thresholded rTMS intensities did not differ in terms of rTMS effects (Beynel et al., 2019).

For tACS, stimulation intensity is not usually individualised and similar to HI-rTMS, the intensity applied varies, usually between 1 and 2 mA. There are not any robust comparison studies that assess the “optimal” intensity for attention tasks, but different intensities could possibly affect outcomes. Perhaps 2 mA was not the optimal intensity for tACS, however, 2 mA has previously induced significant outcomes in cognitive studies (e.g., Kasten and Herrmann, 2017; Kasten et al., 2020). As discussed further in Section “Previous Replication Failures,” there is evidence for intensity-dependent effects in spatial attention (Benwell et al., 2013), but it was not replicated in a follow up study (Learmonth et al., 2015). Studies investigating the biophysics of various tACS intensities could shed light on the interaction between intensity and functional effects. For example, a recent study in non-human primates reported that higher intensities of tACS (comparing 0.5, 1, and 1.5 mA) were able to entrain more cells to induce spike-timing dependent changes, and also increase “burstiness” of neurons (Johnson et al., 2020). Note, this study only looked at short-term stimulation (2 min) so the relevance to longer stimulation used in most tACS studies, offline effects, and functional outcomes are still to be investigated.

Finally, with regards to LI-rTMS, intensity plays a large part in its consideration as a possible new stimulation approach. Animal models using a range of intensities have shown biological and functional effects (Moretti and Rodger, 2022). Our intensity is on the higher range to match with what has been most effective behaviourally in animals (Heath et al., 2018), however, there is still a lot that is unknown about any “optimal” intensity. Future studies to explore dosage parameters and determine the minimum effective intensity, both in humans and animals, would be useful. Since LI-rTMS is subthreshold, we approached the intensity choice in a similar way to tACS and in line with previous LI-rTMS animal models–using a fixed intensity. Part of this reasoning was to remain in line with the animal models and follow a translational pipeline approach, but this differs from convention in HI-rTMS. Although individualising intensity can help normalise stimulation across intra-individual differences in physiological excitability, there are drawbacks with regards to relating thresholded intensities to basic and preclinical research (Turi et al., 2021). Plus, as discussed above, using fixed vs. thresholded intensity approaches do not necessarily predict different rTMS effects (Beynel et al., 2019). In addition, since LI-rTMS remains below the action-potential threshold, individual excitability on the scale of motor or phosphene thresholds are not necessarily relevant to mechanisms of action of LI-rTMS. Similar to tACS, intensity may be important on a cellular level, but using an aggregate measure of cortical excitability is less relevant to LI-rTMS. To be able to step away from the practice of individualised rTMS intensities would make application of LI-rTMS easier and require less expertise for stimulation delivery, more in line with tES approaches. With all of this in consideration, the fixed intensity approach may still be a limitation. Further exploration of dosage-response curves with LI-rTMS comparing the fixed and individualised approaches in the future could help elucidate this.

### Previous Replication Failures

At a group level, previous studies had found that 10 Hz rTMS over the rPPC increased visuospatial attention in the left, contralateral hemispace and increased leftward biases in healthy participants (Kim et al., 2005). Multiple sessions of 10 Hz rTMS also improved hemispatial neglect in stroke patients, when assessed with a line bisection task (Kim et al., 2013). Inhibitory rTMS (1 Hz) also facilitated visuospatial attention in the unilateral hemisphere (e.g., Hilgetag et al., 2001), demonstrating how excitatory and inhibitory stimulation of different hemispheres can effect visuospatial attention in similar ways. Shifts in visuospatial attention have been reported with offline tDCS in a polarity-dependent manner (Sparing et al., 2009; English et al., 2018), online tDCS (Giglia et al., 2011; e.g., Benwell et al., 2015) and online tACS (Schuhmann et al., 2019). Modulation of temporal attention has also been demonstrated with improved attentional blink following TMS over the rPPC (Cooper et al., 2004) and online 20 Hz tACS of frontal and parietal regions (Yaple and Vakhrushev, 2018). Online 10 Hz tACS has also shown evidence for improved temporal discrimination following stimulation on either side of the PPC and leftward shift in spatial bias following rPPC stimulation in a temporal order judgement task (Otsuru et al., 2019).

However, there are also several examples of an absence of cognitive change following brain stimulation. For example, Learmonth et al. (2017), followed up reports of significant modulation of spatial attention in a line bisection task following tDCS seen by Benwell et al. (2015), using a within-subject study design. They were unable to reproduce the same positive results, reporting no significant changes to spatial bias following bi-parietal online tDCS for 15 min. Learmonth et al. (2017) were also unable to replicate an interaction found by Benwell et al. (2015) that a rightward shift in visuospatial attention depended on participant’s baseline task performance and tDCS intensity (1 vs. 2 mA). Similarly, Veniero et al. (2017) ran two experiments assessing tDCS and 10 Hz tACS on spatial attention bias. In their first experiment, they were unable to replicate a shift in spatial attention with cathodal tDCS previously reported by Giglia et al. (2011) and Benwell et al. (2015), but did show significant change in bias during online 10 Hz tACS. However, when they attempted to replicate the 10 Hz tACS experiment in a separate sample using a within subjects design, they were unable to reproduce the shift in spatial attention. When they combined the two experimental samples, the previous 10 Hz tACS result also disappeared with the increase in sample size (Veniero et al., 2017).

Our protocol differed in several ways compared to various online tDCS and tACS experiments detailed above, and therefore is not a direct replication attempt. However, the unreliable nature of brain stimulation-induced cognitive changes, particularly with crossover study designs further underlines the difficulty of interpreting the results of studies that apply new stimulation parameters. Interpretation requires evaluation of the reason behind negative results when using exploratory neurostimulation techniques to better determine whether they reflect a true lack of neuromodulatory effects, or are instead the result of other confounding factors which can underlie unreliable or inconsistent modulatory effects reported in both brain stimulation literature and broader cognitive research (Draheim et al., 2021).

### Limitations–Additional Measures of Individual Variation

Another limit to establishing consistent effects of brain stimulation is the high rate of inter-individual variability. There has been an increasing push to identify predictors and biomarkers that can help predict whether an individual will respond favourably to brain stimulation which could help guide patient or participant selection. For example, functional and structural connectivity have been identified as possible determinants of stimulation effects in individuals. Mariner et al. (2021) showed that at a group level cTBS did not show the expected rightward shift in visuospatial attention with a Landmark test. However, EEG connectivity, specifically connectivity between the rPPC and left temporal-parietal region, was a significant predictor and likely determinant of whether cTBS was able to influence spatial attention on the individual level. Other studies also link inter-individual variability in visuospatial attention following cTBS with changes in functional connectivity and structural connectivity particularly related to the posterior corpus callosum (Schintu et al., 2021). Schintu et al. (2021) discuss the possibility that differences in connectivity change stimulation outcomes due to differential effects on inhibition or excitation of interhemispheric pathways that modulate visuospatial attention (Koch et al., 2011). For example, individuals with more robust callosal pathways may have less effective inhibition of the interhemispheric PPC pathway following cTBS than individuals with weaker connections (Schintu et al., 2021). Therefore an individual’s baseline structural and functional connectivity, which can be influenced by several factors such as sex, genetic, and environmental influences [e.g., training in music (de Manzano and Ullén, 2018) or motor skills (Scholz et al., 2009)] (for review see Lebel and Deoni, 2018) may determine how susceptible they are to stimulation effects.

Availability of γ-aminobutyric acid (GABA) and glutamate, as measured by magnetic resonance spectroscopy, have also been suggested as biomarkers for tDCS effects (Filmer et al., 2019). Training in a response selection task was disrupted by cathodal tDCS over the left the prefrontal cortex. The degree to which training was disrupted was associated with individuals’ concentration of GABA and glutamate in the prefrontal cortex. Individual levels of cortical inhibition, suggested by the ratio between GABA and glutamate concentrations (i.e., more GABA than glutamate), had larger disruptions in task training. The disruption in task training and association with neurochemical availability was only evident with cathodal, not anodal or sham stimulation (Filmer et al., 2019). Interestingly, although they did not assess changes on an individual level, Vidal-Piñeiro et al. (2015) demonstrated that a single stimulation of iTBS, but not cTBS over the left inferior parietal lobe was able to increase GABA concentration in the posterior cingulate cortex, a distal region to the stimulation site. The change in distal GABA concentration and a non-significant change in combined glutamate/glutamine concentration was significantly associated with intrinsic connectivity between inferior parietal lobe and posterior cingulate cortex before TBS. This further suggests that individual functional connectivity modulates brain stimulation effects. Finally, other factors such as genetic variation among plasticity-related genes are also beginning to be explored as contributors to inter-individual differences in brain stimulation responses, e.g., BDNF Val66Met polymorphisms (Cheeran et al., 2008; Abellaneda-Pérez et al., 2022). In sum, multiple levels of variation, down to the genetic level likely influence brain connectivity and consequent responses to brain stimulation, and future work should assess such individual variations in order to attempt to bolster consistency across stimulation studies.

### Future Directions

It may be that cognitive changes assessed solely through experimental tasks were not sensitive enough to pick up on any subtle changes to attention induced by the stimulation. Assessing changes to excitability using motor evoked potentials or EEG may be more suitable as a barometer of whether LI-rTMS can induce changes in humans, and easier to compare quantitatively against HI-rTMS or tES. As demonstrated by Mariner et al. (2021), including EEG can also allow connectivity analysis to be used to further assess determinants behind inter-individual variability, and would allow the ability to use individualised alpha-frequency tACS methods rather than fixed frequency tACS to possibly increase tACS efficacy or after-effects. Comparing effects of online stimulation may also be more likely to produce significant changes and allow more suitable comparison between tES and LI-rTMS, in order to assess the effects of sub-threshold stimulation on cognition and behaviour. However, HI-rTMS is difficult to administer online as it can induce muscle twitching and is accompanied by a loud clicking sound which could distract participants from the task. Due to the exploratory nature of this study in relation to LI-rTMS it may also be that attention was not the most suitable behaviour to assess LI-rTMS effects, although our choice was guided by animal models which have suggested some attention-related effects of LI-rTMS (Poh et al., 2018; Moretti et al., 2021). LI-rTMS may be able to modulate behaviour in other tasks, although it is not yet clear which tasks would be most suitable. For example, it may be that sub-threshold stimulation using LI-rTMS acts under principles of stochastic resonance which was suggested after LI-rTMS modulation of visual evoked potentials in mice (Makowiecki et al., 2018) and is similar to theories proposed for tES (Miniussi et al., 2013). Therefore, perception tasks and inclusion of online LI-rTMS may be a good starting point to look at potential behavioural changes through the lens of optimising signal-to-noise ratio of neural activity.

## Conclusion

This study was the first to assess the effects of LI-rTMS on cognition in humans. LI-rTMS was tolerated extremely well, however, we did not observe any significant changes to spatial or temporal attention. We also did not observe changes to spatial or temporal attention following offline rTMS delivering iTBS and 10 Hz tACS. Since we were unable to modulate attention as has been seen in previous studies using rTMS and tES we cannot yet draw conclusion on how LI-rTMS compares with conventional stimulation currently used in humans and the possible mechanisms underlying these techniques. Our null results following HI-rTMS and tACS provide evidence supporting ineffective modulation of attention when applying iTBS and offline tACS.

## Data Availability Statement

The raw data supporting the conclusions of this article will be made available by the authors, without undue reservation.

## Ethics Statement

The studies involving human participants were reviewed and approved by the University of Western Australia Human Research Ethics Committee. The patients/participants provided their written informed consent to participate in this study.

## Author Contributions

JM: conceptualisation, methodology, writing–original draft, review and editing, visualisation, formal analysis, investigation, and software. WM: writing–review and editing, resources, and formal analysis. AH: writing–review and editing and supervision. JR and TV: writing–review and editing, supervision, conceptualisation, and methodology. All authors contributed to the article and approved the submitted version.

## Conflict of Interest

The authors declare that the research was conducted in the absence of any commercial or financial relationships that could be construed as a potential conflict of interest.

## Publisher’s Note

All claims expressed in this article are solely those of the authors and do not necessarily represent those of their affiliated organizations, or those of the publisher, the editors and the reviewers. Any product that may be evaluated in this article, or claim that may be made by its manufacturer, is not guaranteed or endorsed by the publisher.

## References

[B1] Abellaneda-Pérez K. Martin-Trias P. Cassé-Perrot C. Vaqué-Alcázar L. Lanteaume L. Solana E. (2022). BDNF Val66Met gene polymorphism modulates brain activity following rTMS-induced memory impairment. *Sci. Rep.* 12:176. 10.1038/s41598-021-04175-x 34997117 PMC8741781

[B2] Antal A. Alekseichuk I. Bikson M. Brockmöller J. Brunoni A. R. Chen R. (2017). Low intensity transcranial electric stimulation: safety, ethical, legal regulatory and application guidelines. *Clin. Neurophysiol.* 128 1774–1809. 10.1016/j.clinph.2017.06.001 28709880 PMC5985830

[B3] Arasanz C. P. Staines W. R. Schweizer T. A. (2012). Isolating a cerebellar contribution to rapid visual attention using transcranial magnetic stimulation. *Front. Behav. Neurosci.* 6:55. 10.3389/fnbeh.2012.00055 22936903 PMC3426766

[B4] Benwell C. S. Y. Learmonth G. Miniussi C. Harvey M. Thut G. (2015). Non-linear effects of transcranial direct current stimulation as a function of individual baseline performance: evidence from biparietal tDCS influence on lateralized attention bias. *Cortex* 69 152–165. 10.1016/j.cortex.2015.05.007 26073146

[B5] Benwell C. S. Y. Thut G. Learmonth G. Harvey M. (2013). Spatial attention: differential shifts in pseudoneglect direction with time-on-task and initial bias support the idea of observer subtypes. *Neuropsychologia* 51 2747–2756. 10.1016/j.neuropsychologia.2013.09.030 24076376

[B6] Beynel L. Appelbaum L. Luber B. Crowell C. Hilbig S. Lim W. (2019). Effects of online repetitive transcranial magnetic stimulation (rTMS) on cognition: a meta-analysis and recommendations for future studies. *Brain Stimul.* 12:565. 10.1016/j.brs.2018.12.870 PMC7654714 31473301

[B7] Boroojerdi B. Meister I. G. Foltys H. Sparing R. Cohen L. G. Töpper R. (2002). Visual and motor cortex excitability: a transcranial magnetic stimulation study. *Clin. Neurophysiol.* 113 1501–1504. 10.1016/S1388-2457(02)00198-0 12169333

[B8] Cazzoli D. Rosenthal C. R. Kennard C. Zito G. A. Hopfner S. Müri R. M. (2015). Theta burst stimulation improves overt visual search in spatial neglect independently of attentional load. *Cortex* 73 317–329. 10.1016/j.cortex.2015.09.009 26547867

[B9] Cazzoli D. Wurtz P. Müri R. M. Hess C. W. Nyffeler T. (2009). Interhemispheric balance of overt attention: a theta burst stimulation study. *Eur. J. Neurosci.* 29 1271–1276. 10.1111/j.1460-9568.2009.06665.x 19302162

[B10] Chechlacz M. Humphreys G. W. Sotiropoulos S. N. Kennard C. Cazzoli D. (2015). Structural organization of the corpus callosum predicts attentional shifts after continuous theta burst stimulation. *J. Neurosci.* 35 15353–15368. 10.1523/JNEUROSCI.2610-15.2015 26586822 PMC4649006

[B11] Cheeran B. Talelli P. Mori F. Koch G. Suppa A. Edwards M. (2008). A common polymorphism in the brain-derived neurotrophic factor gene (BDNF) modulates human cortical plasticity and the response to rTMS. *J. Physiol.* 586 5717–5725. 10.1113/jphysiol.2008.159905 18845611 PMC2655403

[B12] Clayton M. S. Yeung N. Cohen Kadosh R. (2015). The roles of cortical oscillations in sustained attention. *Trends Cogn. Sci.* 19 188–195. 10.1016/j.tics.2015.02.004 25765608

[B13] Clayton M. S. Yeung N. Kadosh R. C. (2019). Electrical stimulation of alpha oscillations stabilizes performance on visual attention tasks. *J. Exp. Psychol. Gen.* 148 203–220. 10.1037/xge0000502 30421943

[B14] Cooper A. C. G. Humphreys G. W. Hulleman J. Praamstra P. Georgeson M. (2004). Transcranial magnetic stimulation to right parietal cortex modifies the attentional blink. *Exp. Brain Res.* 155 24–29. 10.1007/s00221-003-1697-9 15064881

[B15] Dayan E. Censor N. Buch E. R. Sandrini M. Cohen L. G. (2013). Noninvasive brain stimulation: from physiology to network dynamics and back. *Nat. Neurosci.* 16 838–844. 10.1038/nn.3422 23799477 PMC4876726

[B16] de Manzano Ö. Ullén F. (2018). Same genes, different brains: neuroanatomical differences between monozygotic twins discordant for musical training. *Cereb. Cortex* 28 387–394. 10.1093/cercor/bhx299 29136105

[B17] Demeter E. (2016). Enhancing cognition with theta burst stimulation. *Curr. Behav. Neurosci. Rep.* 3 87–94. 10.1007/s40473-016-0072-7

[B18] Draheim C. Tsukahara J. S. Martin J. D. Mashburn C. A. Engle R. W. (2021). A toolbox approach to improving the measurement of attention control. *J. Exp. Psychol. Gen.* 150 242–275. 10.1037/xge0000783 32700925

[B19] English M. C. W. Kitching E. S. Maybery M. T. Visser T. A. W. (2018). Modulating attentional biases of adults with autistic traits using transcranial direct current stimulation: a pilot study. *Autism Res.* 11 385–390. 10.1002/aur.1895 29155494

[B20] Esterman M. Thai M. Okabe H. DeGutis J. Saad E. Laganiere S. E. (2017). Network-targeted cerebellar transcranial magnetic stimulation improves attentional control. *Neuroimage* 156 190–198. 10.1016/j.neuroimage.2017.05.011 28495634 PMC5973536

[B21] Fierro B. Brighina F. Oliveri M. Piazza A. La Bua V. Buffa D. (2000). Contralateral neglect induced by right posterior parietal rTMS in healthy subjects. *Neuroreport* 11 1519–1521. 10.1097/00001756-200005150-00031 10841369

[B22] Filmer H. L. Dux P. E. Mattingley J. B. (2015). Dissociable effects of anodal and cathodal tDCS reveal distinct functional roles for right parietal cortex in the detection of single and competing stimuli. *Neuropsychologia* 74 120–126. 10.1016/j.neuropsychologia.2015.01.038 25637773

[B23] Filmer H. L. Ehrhardt S. E. Bollmann S. Mattingley J. B. Dux P. E. (2019). Accounting for individual differences in the response to tDCS with baseline levels of neurochemical excitability. *Cortex* 115 324–334. 10.1016/j.cortex.2019.02.012 30903834

[B24] Flöel A. (2014). tDCS-enhanced motor and cognitive function in neurological diseases. *Neuroimage* 85 934–947. 10.1016/j.neuroimage.2013.05.098 23727025

[B25] Foxe J. J. Snyder A. C. (2011). The role of alpha-band brain oscillations as a sensory suppression mechanism during selective attention. *Front. Psychol.* 2:154. 10.3389/fpsyg.2011.00154 21779269 PMC3132683

[B26] Fu W. Song W. Zhang Y. Yang Y. Huo S. Zhang R. (2015). Long-term effects of continuous theta-burst stimulation in visuospatial neglect. *J. Int. Med. Res.* 43 196–203. 10.1177/0300060513498663 25589237

[B27] Gamboa O. L. Antal A. Moliadze V. Paulus W. (2010). Simply longer is not better: reversal of theta burst after-effect with prolonged stimulation. *Exp. Brain Res.* 204 181–187. 10.1007/s00221-010-2293-4 20567808 PMC2892066

[B28] Gan T. Nikolin S. Loo C. K. Martin D. M. (2019). Effects of high-definition transcranial direct current stimulation and theta burst stimulation for modulating the posterior parietal cortex. *J. Int. Neuropsychol. Soc.* 25 972–984. 10.1017/S1355617719000766 31397255

[B29] Giglia G. Mattaliano P. Puma A. Rizzo S. Fierro B. Brighina F. (2011). Neglect-like effects induced by tDCS modulation of posterior parietal cortices in healthy subjects. *Brain Stimul.* 4 294–299. 10.1016/j.brs.2011.01.003 22032745

[B30] Heath A. Lindberg D. R. Makowiecki K. Gray A. Asp A. J. Rodger J. (2018). Medium- and high-intensity rTMS reduces psychomotor agitation with distinct neurobiologic mechanisms. *Transl. Psychiatry* 8:126. 10.1038/s41398-018-0129-3 29976924 PMC6033856

[B31] Hilgetag C. C. Théoret H. Pascual-Leone A. (2001). Enhanced visual spatial attention ipsilateral to rTMS-induced “virtual lesions” of human parietal cortex. *Nat. Neurosci.* 4 953–957. 10.1038/nn0901-953 11528429

[B32] Hopfinger J. B. Parsons J. Fröhlich F. (2017). Differential effects of 10-Hz and 40-Hz transcranial alternating current stimulation (tACS) on endogenous versus exogenous attention. *Cogn. Neurosci.* 8 102–111. 10.1080/17588928.2016.1194261 27297977

[B33] Huang Y.-Z. Edwards M. J. Rounis E. Bhatia K. P. Rothwell J. C. (2005). Theta burst stimulation of the human motor cortex. *Neuron* 45 201–206. 10.1016/j.neuron.2004.12.033 15664172

[B34] Jensen O. Mazaheri A. (2010). Shaping functional architecture by oscillatory alpha activity: gating by inhibition. *Front. Hum. Neurosci.* 4:186. 10.3389/fnhum.2010.00186 21119777 PMC2990626

[B35] Johnson L. Alekseichuk I. Krieg J. Doyle A. Yu Y. Vitek J. (2020). Dose-dependent effects of transcranial alternating current stimulation on spike timing in awake nonhuman primates. *Sci. Adv.* 6:eaaz2747. 10.1126/sciadv.aaz2747 32917605 PMC7467690

[B36] Kammer T. Beck S. Erb M. Grodd W. (2001). The influence of current direction on phosphene thresholds evoked by transcranial magnetic stimulation. *Clin. Neurophysiol.* 112 2015–2021. 10.1016/S1388-2457(01)00673-3 11682339

[B37] Kasten F. H. Herrmann C. S. (2017). Transcranial alternating current stimulation (tACS) enhances mental rotation performance during and after stimulation. *Front. Hum. Neurosci.* 11:2. 10.3389/fnhum.2017.00002 28197084 PMC5281636

[B38] Kasten F. H. Wendeln T. Stecher H. I. Herrmann C. S. (2020). Hemisphere-specific, differential effects of lateralized, occipital–parietal α- versus γ-tACS on endogenous but not exogenous visual-spatial attention. *Sci. Rep.* 10:12270. 10.1038/s41598-020-68992-2 32703961 PMC7378174

[B39] Kim B. R. Chun M. H. Kim D. Y. Lee S. J. (2013). Effect of high- and low-frequency repetitive transcranial magnetic stimulation on visuospatial neglect in patients with acute stroke: a double-blind, sham-controlled trial. *Arch. Phys. Med. Rehabil.* 94 803–807. 10.1016/j.apmr.2012.12.016 23298790

[B40] Kim Y. H. Min S. J. Ko M. H. Park J. W. Sung H. J. Lee P. K. W. (2005). Facilitating visuospatial attention for the contralateral hemifield by repetitive TMS on the posterior parietal cortex. *Neurosci. Lett.* 382 280–285. 10.1016/j.neulet.2005.03.043 15925104

[B41] Klink K. Paßmann S. Kasten F. H. Peter J. (2020). The modulation of cognitive performance with transcranial alternating current stimulation: a systematic review of frequency-specific effects. *Brain Sci.* 10 1–33. 10.3390/brainsci10120932 33276533 PMC7761592

[B42] Koch G. Cercignani M. Bonnì S. Giacobbe V. Bucchi G. Versace V. (2011). Asymmetry of parietal interhemispheric connections in humans. *J. Neurosci.* 31 8967–8975. 10.1523/JNEUROSCI.6567-10.2011 21677180 PMC6622945

[B43] Kuo M.-F. Nitsche M. A. (2012). Effects of transcranial electrical stimulation on cognition. *Clin. EEG Neurosci.* 43 192–199. 10.1177/1550059412444975 22956647

[B44] Learmonth G. Felisatti F. Siriwardena N. Checketts M. Benwell C. S. Y. Märker G. (2017). No interaction between tDCS current strength and baseline performance: a conceptual replication. *Front. Neurosci.* 11:664. 10.3389/fnins.2017.00664 29249932 PMC5717015

[B45] Learmonth G. Gallagher A. Gibson J. Thut G. Harvey M. (2015). Intra-and inter-task reliability of spatial attention measures in pseudoneglect. *PLoS One* 10:e0138379. 10.1371/journal.pone.0138379 26378925 PMC4574708

[B46] Lebel C. Deoni S. (2018). The development of brain white matter microstructure. *Neuroimage* 182 207–218. 10.1016/j.neuroimage.2017.12.097 29305910 PMC6030512

[B47] Lefaucheur J.-P. Aleman A. Baeken C. Benninger D. H. Brunelin J. Di Lazzaro V. (2020). Evidence-based guidelines on the therapeutic use of repetitive transcranial magnetic stimulation (rTMS): an update (2014–2018). *Clin. Neurophysiol.* 125 2150–2206. 10.1016/j.clinph.2019.11.002 25034472

[B48] Lenz M. Platschek S. Priesemann V. Becker D. Willems L. M. Ziemann U. (2015). Repetitive magnetic stimulation induces plasticity of excitatory postsynapses on proximal dendrites of cultured mouse CA1 pyramidal neurons. *Brain Struct. Funct.* 220 3323–3337. 10.1007/s00429-014-0859-9 25108309

[B49] Loftus A. M. Nicholls M. E. R. (2012). Testing the activation-orientation account of spatial attentional asymmetries using transcranial direct current stimulation. *Neuropsychologia* 50 2573–2576. 10.1016/j.neuropsychologia.2012.07.003 22820341

[B50] Luber B. Lisanby S. H. (2014). Enhancement of human cognitive performance using transcranial magnetic stimulation (TMS). *Neuroimage* 85 961–970. 10.1016/j.neuroimage.2013.06.007 23770409 PMC4083569

[B51] Makowiecki K. Garrett A. Harvey A. R. Rodger J. (2018). Low-intensity repetitive transcranial magnetic stimulation requires concurrent visual system activity to modulate visual evoked potentials in adult mice. *Sci. Rep.* 8:5792. 10.1038/s41598-018-23979-y 29643395 PMC5895738

[B52] Mariner J. Loetscher T. Hordacre B. (2021). Parietal cortex connectivity as a marker of shift in spatial attention following continuous theta burst stimulation. *Front. Hum. Neurosci.* 15:718662. 10.3389/fnhum.2021.718662 34566602 PMC8455944

[B53] Martiny K. Lunde M. Bech P. (2010). Transcranial low voltage pulsed electromagnetic fields in patients with treatment-resistant depression. *Biol. Psychiatry* 68 163–169. 10.1016/j.biopsych.2010.02.017 20385376

[B54] Milner A. D. Brechmann M. Pagliarini L. (1992). To halve and to halve not: an analysis of line bisection judgements in normal subjects. *Neuropsychologia* 30 515–526. 10.1016/0028-3932(92)90055-Q 1641116

[B55] Miniussi C. Harris J. A. Ruzzoli M. (2013). Modelling non-invasive brain stimulation in cognitive neuroscience. *Neurosci. Biobehav. Rev.* 37 1702–1712. 10.1016/j.neubiorev.2013.06.014 23827785

[B56] Moretti J. Poh E. Z. Bolland S. J. Harvey A. R. Albrecht M. A. Rodger J. (2021). Concurrent LI-rTMS induces changes in c-Fos expression but not behavior during a progressive ratio task with adult ephrin-A2A5−/− mice. *Behav. Brain Res.* 400:113011. 10.1016/j.bbr.2020.113011 33181182

[B57] Moretti J. Rodger J. (2022). A little goes a long way: neurobiological effects of low intensity rTMS and implications for mechanisms of rTMS. *Curr. Res. Neurobiol.* 3:100033. 10.1016/j.crneur.2022.100033 PMC9846462 36685761

[B58] Nyffeler T. Cazzoli D. Hess C. W. Müri R. M. (2009). One session of repeated parietal theta burst stimulation trains induces long-lasting improvement of visual neglect. *Stroke* 40 2791–2796. 10.1161/STROKEAHA.109.552323 19520986

[B59] Nyffeler T. Cazzoli D. Wurtz P. Lüthi M. Von Wartburg R. Chaves S. (2008). Neglect-like visual exploration behaviour after theta burst transcranial magnetic stimulation of the right posterior parietal cortex. *Eur. J. Neurosci.* 27 1809–1813. 10.1111/j.1460-9568.2008.06154.x 18371083

[B60] Otsuru N. Kamijo K. Otsuki T. Kojima S. Miyaguchi S. Saito K. (2019). 10 Hz transcranial alternating current stimulation over posterior parietal cortex facilitates tactile temporal order judgment. *Behav. Brain Res.* 368:111899. 10.1016/j.bbr.2019.111899 30978408

[B61] Peirce J. Gray J. R. Simpson S. MacAskill M. Höchenberger R. Sogo H. (2019). PsychoPy2: experiments in behavior made easy. *Behav. Res. Methods* 51 195–203. 10.3758/s13428-018-01193-y 30734206 PMC6420413

[B62] Poh E. Z. Harvey A. R. Makowiecki K. Rodger J. (2018). Online LI-rTMS during a visual learning task : differential impacts on visual circuit and behavioural plasticity in adult Ephrin-A2A5 Mice. *eNeuro* 5:ENEURO.0163-17.2018. 10.1523/ENEURO.0163-17.2018 29464193 PMC5815844

[B63] Posner M. I. Petersen S. E. (1990). The attention system of the human brain. *Annu. Rev. Neurosci.* 13 25–42. 10.1146/annurev.ne.13.030190.000325 2183676

[B64] Reteig L. C. Talsma L. J. van Schouwenburg M. R. Slagter H. A. (2017). Transcranial electrical stimulation as a tool to enhance attention. *J. Cogn. Enhanc.* 1 10–25. 10.1007/s41465-017-0010-y

[B65] Rizk S. Ptak R. Nyffeler T. Schnider A. Guggisberg A. G. (2013). Network mechanisms of responsiveness to continuous theta-burst stimulation. *Eur. J. Neurosci.* 38 3230–3238. 10.1111/ejn.12334 23941616

[B66] Rohan M. Parow A. Stoll A. L. Demopulos C. Friedman S. Ph D. (2004). Low-field magnetic stimulation in bipolar depression using a MRI-based stimulator. *Am. J. Psychiatry* 161 93–98. 10.1176/appi.ajp.161.1.93 14702256

[B67] Rohan M. L. Yamamoto R. T. Ravichandran C. T. Cayetano K. R. Morales O. G. Olson D. P. (2014). Rapid mood-elevating effects of low field magnetic stimulation in depression. *Biol. Psychiatry* 76 186–193. 10.1016/j.biopsych.2013.10.024 24331545

[B68] Rossi S. Hallett M. Rossini P. M. Pascual-Leone A. Avanzini G. Bestmann S. (2009). Safety, ethical considerations, and application guidelines for the use of transcranial magnetic stimulation in clinical practice and research. *Clin. Neurophysiol.* 120 2008–2039. 10.1016/j.clinph.2009.08.016 19833552 PMC3260536

[B69] Sack A. T. Kadosh R. C. Schuhmann T. Moerel M. Walsh V. Goebel R. (2009). Optimizing functional accuracy of TMS in cognitive studies: a comparison of methods. *J. Cogn. Neurosci.* 21 207–221. 10.1162/jocn.2009.21126 18823235

[B70] Santarnecchi E. Brem A. K. Levenbaum E. Thompson T. Kadosh R. C. Pascual-Leone A. (2015). Enhancing cognition using transcranial electrical stimulation. *Curr. Opin. Behav. Sci.* 4 171–178. 10.1016/j.cobeha.2015.06.003

[B71] Schintu S. Cunningham C. A. Freedberg M. Taylor P. Gotts S. J. Shomstein S. (2021). Callosal anisotropy predicts attentional network changes after parietal inhibitory stimulation. *Neuroimage* 226:117559. 10.1016/j.neuroimage.2020.117559 33189929 PMC7885523

[B72] Scholz J. Klein M. C. Behrens T. E. J. Johansen-Berg H. (2009). Training induces changes in white-matter architecture. *Nat. Neurosci.* 12 1370–1371. 10.1038/nn.2412 19820707 PMC2770457

[B73] Schuhmann T. Kemmerer S. K. Duecker F. de Graaf T. A. ten Oever S. De Weerd P. (2019). Left parietal tACS at alpha frequency induces a shift of visuospatial attention. *PLoS One* 14:e0217729. 10.1371/journal.pone.0217729 31774818 PMC6881009

[B74] Sparing R. Thimm M. Hesse M. D. Küst J. Karbe H. Fink G. R. (2009). Bidirectional alterations of interhemispheric parietal balance by non-invasive cortical stimulation. *Brain* 132 3011–3020. 10.1093/brain/awp154 19528092

[B75] Stewart L. M. Walsh V. Rothwell J. C. (2001). Motor and phosphene thresholds: a transcranial magnetic stimulation correlation study. *Neuropsychologia* 39 415–419. 10.1016/S0028-3932(00)00130-5 11164880

[B76] Thut G. Nietzel A. Pascual-Leone A. (2005). Dorsal posterior parietal rTMS affects voluntary orienting of visuospatial attention. *Cereb. Cortex* 15 628–638. 10.1093/cercor/bhh164 15342434

[B77] Turi Z. Lenz M. Paulus W. Mittner M. Vlachos A. (2021). Selecting stimulation intensity in repetitive transcranial magnetic stimulation studies: a systematic review between 1991 and 2020. *Eur. J. Neurosci.* 53 3404–3415. 10.1111/ejn.15195 33754397

[B78] Varnava A. Dervinis M. Chambers C. D. (2013). The predictive nature of pseudoneglect for visual neglect: evidence from parietal theta burst stimulation. *PLoS One* 8:e65851. 10.1371/journal.pone.0065851 23823975 PMC3688802

[B79] Veniero D. Benwell C. S. Y. Ahrens M. M. Thut G. (2017). Inconsistent effects of parietal α-tACS on Pseudoneglect across two experiments: a failed internal replication. *Front. Psychol.* 8:952. 10.3389/fpsyg.2017.00952 28642729 PMC5463322

[B80] Vidal-Piñeiro D. Martín-Trias P. Falcón C. Bargalló N. Clemente I. C. Valls-solé J. (2015). Neurochemical modulation in posteromedial default-mode network cortex induced by transcranial magnetic stimulation. *Brain Stimul.* 8 937–944. 10.1016/j.brs.2015.04.005 25981159

[B81] Vossen A. Gross J. Thut G. (2015). Alpha power increase after transcranial alternating current stimulation at alpha frequency (a-tACS) reflects plastic changes rather than entrainment. *Brain Stimul.* 8 499–508. 10.1016/j.brs.2014.12.004 25648377 PMC4464304

[B82] Whybird M. Coats R. Vuister T. Harrison S. Booth S. Burke M. (2021). The role of the posterior parietal cortex on cognition: an exploratory study. *Brain Res.* 1764 147452. 10.1016/j.brainres.2021.147452 33838128

[B83] Wischnewski M. Schutter D. J. L. G. (2015). Efficacy and time course of theta burst stimulation in healthy humans. *Brain Stimul.* 8 685–692. 10.1016/j.brs.2015.03.004 26014214

[B84] Wu X. Wang L. Geng Z. Wei L. Yan Y. Xie C. (2021). Improved cognitive promotion through accelerated magnetic stimulation. *eNeuro* 8:ENEURO.0392-20.2020. 10.1523/eneuro.0392-20.2020 33452108 PMC7901150

[B85] Yang W. Liu T.-T. Song X.-B. Zhang Y. Li Z.-H. Cui Z.-H. (2015). Comparison of different stimulation parameters of repetitive transcranial magnetic stimulation for unilateral spatial neglect in stroke patients. *J. Neurol. Sci.* 359 219–225. 10.1016/j.jns.2015.08.1541 26671118

[B86] Yaple Z. Vakhrushev R. (2018). Modulation of the frontal-parietal network by low intensity anti-phase 20 Hz transcranial electrical stimulation boosts performance in the attentional blink task. *Int. J. Psychophysiol.* 127 11–16. 10.1016/j.ijpsycho.2018.02.014 29499241

